# Supplementary material for: Viral load suppression and HIV-1 drug resistance mutations in persons with HIV on TLD/TAFED in Zambia
Source: PLoS One. 2024 Sep 6;19(9):e0308869. doi: 10.1371/journal.pone.0308869 (PMC11379217; doi:10.1371/journal.pone.0308869)
Supplement: Attachment — Submitted filename: For Maria to review.pdf [file pone.0308869.s006.pdf]

PLOS ONE

Commented [MN1]:

Viral load suppression and HIV-1 drug resistance mutations in persons with HIV on TLD/TAFED in Zambia: A cross-sectional study

--Manuscript Draft--

**Manuscript Number:**	PONE-D-24-12439
**Article Type:**	Research Article
**Full Title:**	Viral load suppression and HIV-1 drug resistance mutations in persons with HIV on TLD/TAFED in Zambia: A cross-sectional study
**Short Title:**	Viral load suppression and HIV-1 drug resistance in persons with HIV on TLD/TAFED in Zambia
**Corresponding Author:**	Emmanuel Luhamba Luwaya, BSc, MSc<br>Mulungushi University<br>Livingstone, ZAMBIA
**Keywords:**	Viral load; HIV drug resistance mutations; TLD/TAFED
**Abstract:**	Background: An increase in the prevalence of HIV drug resistance (HIVDR) has been reported in recent years, especially in persons on non-nucleoside reverse transcriptase inhibitors (NNRTIs) due to their low genetic barrier to mutations. However, there is a paucity of epidemiological data quantifying HIVDR in the era of new drugs like dolutegravir (DTG) in sub-Saharan Africa. We, therefore, sought to determine the prevalence and correlates of viral load (VL) suppression in adult people with HIV (PWH) on a fixed-dose combination of tenofovir disoproxil fumarate/lamivudine/dolutegravir (TLD) or tenofovir alafenamide/emtricitabine/dolutegravir (TAFED) and describe patterns of mutations in individuals failing treatment.<br>Methods: We conducted a cross-sectional study among 384 adults living with HIV aged ≥15 years between May 2023 and August 2023. Demographic, laboratory and clinical data were collected from electronic health records using a data collection form. Viral load suppression was defined as plasma HIV-1 RNA VL of <1000 copies/ml after being on ART for ≥ 6 months. SPSS version 22 to analyze the data. Descriptive statistics and logistic regression were the statistical methods used.<br>Results: The median (interquartile range (IQR)) age was 22 (IQR 18, 38) years, and 66.1% (n=254) were females. VL suppression was 90.9% (n=349); (95% confidence interval (CI) 87.6%-93.6%) after switching to TLD/TAFED. Among the virally suppressed, the majority (67%, n=234) were female. Those who missed ≥2 doses in the last 30 days prior to the most recent review were less likely to attain viral suppression compared to those who did not miss any dose (adjusted odds ratio (AOR) 0.075; 95% CI 0.028-0.199; p<0.001), whereas those who were on a protease inhibitor (PI)-based regimen for at least 6 months before switching to TLD/TAFED were also less likely to attain viral suppression compared to those who were on NNRTI-based regimen for at least 6 months before switching; AOR 0.193 (95% CI 0.057-0.652; p=0.008. Four participants had resistance mutations to lamivudine and tenofovir. The most common NRTI mutations were M184MV and K65R while K101E was the most common NNRTI mutation.<br><br>Conclusion: Our findings show that viral suppression was high after switching to TLD/TAFED; but lower than the last 95 target of UNAIDS. Adherence to, and type of antiretroviral therapy used before switching to TLD/TAFED were significant correlates of VL suppression. We, therefore, recommend prompt switching of PWH to TLD/TAFED regimen and close monitoring to enhance adherence to therapy.
**Order of Authors:**	Emmanuel Luhamba Luwaya, BSc, MSc
	Lackson Mwape, BSc, MSc
	Kaole Bwalya, BSc, MSc

Powered by Editorial Manager® and ProduXion Manager® from Aries Systems Corporation

	Chileleko Siakabanze, BSc, MSc
	Benson Malambo Hamooya, BSc, MSc, PhD

Powered by Editorial Manager® and ProduXion Manager® from Aries Systems Corporation

	Sepiso K. Masenga, BSc, MSc, PhD
**Opposed Reviewers:**	
**Additional Information:**	
**Question**	**Response**
**Financial Disclosure**<br><br>Enter a financial disclosure statement that describes the sources of funding for the work included in this submission. Review the submission guidelines for detailed requirements. View published research articles from *PLOS ONE* for specific examples.<br><br>This statement is required for submission and **will appear in the published article** if the submission is accepted. Please make sure it is accurate.<br><br>**Funded studies**<br>Enter a statement with the following details:<br>• Initials of the authors who received each award<br>• Grant numbers awarded to each author<br>• The full name of each funder<br>• URL of each funder website<br>• Did the sponsors or funders play any role in the study design, data collection and analysis, decision to publish, or preparation of the manuscript?<br><br>Did you receive funding for this work?	The author(s) received no specific funding for this work.
**Competing Interests**<br><br>Use the instructions below to enter a competing interest statement for this submission. On behalf of all authors, disclose any competing interests that could be perceived to bias this work—acknowledging all financial supportand any other relevant financial or non-financial competing interests.<br><br>This statement is required for submission and **will appear in the published article** if	The authors have declared that no competing interests exist.

the submission is accepted. Please make sure it is accurate and that any funding sources listed in your Funding Information later in the submission form are also declared in your Financial Disclosure statement.<br><br>View published research articles from *PLOS ONE* for specific examples.<br><br>**NO authors have competing interests**<br><br>Enter: *The authors have declared that no competing interests exist.*<br><br>**Authors with competing interests**<br><br>Enter competing interest details beginning with this statement:<br><br>*I have read the journal's policy and the authors of this manuscript have the following competing interests: [insert competing interests here]*<br><br>* typeset	
**Ethics Statement**<br><br>Enter an ethics statement for this submission. This statement is required if the study involved:<br><br>• Human participants<br>• Human specimens or tissue<br>• Vertebrate animals or cephalopods<br>• Vertebrate embryos or tissues<br>• Field research<br><br>Write "N/A" if the submission does not require an ethics statement.<br><br>General guidance is provided below. Consult the submission guidelines for detailed instructions. **Make sure that all information entered here is included in the Methods section of the manuscript.**	Ethical approval was obtained from Mulungushi University Health Sciences Research Ethics Committee (MUHSREC) (Assurance No. FWA0002888 IRB00012281 of IORG0010344 on the 5th of April, 2023 and the National Health Research Authority (NHRA) on the 11th May, 2023. Permission to conduct research was given by Arthur Davison children's hospital administration. Written consent was waived by the ethics committee due to the retrospective nature of the study where secondary data was used. No personal identifying data were obtained such that it was not possible to identify participants.

Powered by Editorial Manager® and ProduXion Manager® from Aries Systems Corporation

**Format for specific study types**<br><br>**Human Subject Research (involving human participants and/or tissue)**<br>• Give the name of the institutional review board or ethics committee that approved the study<br>• Include the approval number and/or a statement indicating approval of this research<br>• Indicate the form of consent obtained (written/oral) or the reason that consent was not obtained (e.g. the data were analyzed anonymously)<br><br>**Animal Research (involving vertebrate animals, embryos or tissues)**<br>• Provide the name of the Institutional Animal Care and Use Committee (IACUC) or other relevant ethics board that reviewed the study protocol, and indicate whether they approved this research or granted a formal waiver of ethical approval<br>• Include an approval number if one was obtained<br>• If the study involved *non-human primates*, add *additional details* about animal welfare and steps taken to ameliorate suffering<br>• If anesthesia, euthanasia, or any kind of animal sacrifice is part of the study, include briefly which substances and/or methods were applied<br><br>**Field Research**<br><br>Include the following details if this study involves the collection of plant, animal, or other materials from a natural setting:<br>• Field permit number<br>• Name of the institution or relevant body that granted permission	
**Data Availability**<br><br>Authors are required to make all data underlying the findings described fully available, without restriction, and from the time of publication. PLOS allows rare exceptions to address legal and ethical concerns. See the PLOS Data Policy and FAQ for detailed information.	Yes - all data are fully available without restriction

Powered by Editorial Manager® and ProduXion Manager® from Aries Systems Corporation

A Data Availability Statement describing where the data can be found is required at submission. Your answers to this question constitute the Data Availability Statement and **will be published in the article**, if accepted.<br><br>**Important:** Stating 'data available on request from the author' is not sufficient. If your data are only available upon request, select 'No' for the first question and explain your exceptional situation in the text box.<br><br>Do the authors confirm that all data underlying the findings described in their manuscript are fully available without restriction?	
**Describe where the data may be found in full sentences. If you are copying our sample text, replace any instances of XXX with the appropriate details.**<br><br>• If the data are **held or will be held in a public repository**, include URLs, accession numbers or DOIs. If this information will only be available after acceptance, indicate this by ticking the box below. For example: *All XXX files are available from the XXX database (accession number(s) XXX, XXX.).*<br>• If the data are all contained **within the manuscript and/or Supporting Information files**, enter the following: *All relevant data are within the manuscript and its Supporting Information files.*<br>• If neither of these applies but you are able to provide **details of access elsewhere**, with or without limitations, please do so. For example:<br><br>*Data cannot be shared publicly because of [XXX]. Data are available from the XXX Institutional Data Access / Ethics Committee (contact via XXX) for researchers who meet the criteria for access to confidential data.*<br><br>*The data underlying the results presented in the study are available from (include the name of the third party*	All relevant data are within the manuscript and its supporting information files.

and contact information or URL).<br>• This text is appropriate if the data are owned by a third party and authors do not have permission to share the data.<br><br>* typeset	
Additional data availability information:	Tick here if the URLs/accession numbers/DOIs will be available only after acceptance of the manuscript for publication so that we can ensure their inclusion before publication.; Tick here if your circumstances are not covered by the questions above and you need the journal's help to make your data available.

Cover Letter

Luwaya Emmanuel. L

School of Medicine and Health Sciences

Mulungushi University

P.O Box 60009, Livingstone campus, Zambia

luwayaemmanuel@gmail.com

27/03/2023

The Editor,

PLOS ONE Journal.

Dear Editor,

RE: **Manuscript Submission for Review –"Viral load suppression and HIV-1 drug resistance mutations in persons with HIV on TLD/TAFED in Zambia"**

I am pleased to submit our manuscript entitled **"Viral load suppression and HIV-1 drug resistance mutations in persons with HIV on TLD/TAFED in Zambia"** for your consideration for publication in PLOS ONE.

This manuscript presents findings of a cross-sectional study conducted in Ndola, Zambia at Arthur Davison Children's Hospital in persons living with HIV (PLWH). In this study, we sought to determine the prevalence and correlates of viral load (VL) suppression in adult PLWH on a fixed-dose combination of tenofovir disoproxil fumarate/lamivudine/dolutegravir (TLD) or tenofovir alafenamide/emtricitabine/dolutegravir (TAFED) and describe patterns of mutations in individuals failing treatment.

We believe that our study is valuable and adds to the knowledge in HIV research will be significant in identifying factors that will help attain 95%-95%-95% target. Our study indicates that adherence is a significant factor in attaining viral suppression. Proper methods of monitoring adherence will be vital in helping most PLWH attain viral suppression.

Please find attached the manuscript, tables, figures, and supplementary materials. We affirm that this work has not been submitted elsewhere for publication, and all authors have given their approval for the submission.

Thank you for your time and consideration. We eagerly anticipate the opportunity to contribute to PLOS ONE's body of knowledge.

Sincerely,

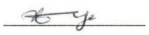

Luwaya Emmanuel. L. MSc,

Corresponding Author

HAND Research Group, School of Medicine and Health Sciences

Mulungushi University, Livingstone campus, Zambia

Email: luwayaemmanuel@gmail.com

Mobile: +260963326481

Manuscript Click here to access/download;Manuscript;Manuscript.docx

Viral load suppression and HIV-1 drug resistance mutations in persons with HIV on TLD/TAFED in Zambia

Emmanuel L. Luwaya[1], Lackson Mwape[1], Kaole Bwalya[1], Chileleko Siakabanze[1], Benson M. Hamooya[1], Sepiso K. Masenga[1,2]

[1]Mulungushi University, School of Medicine and Health Sciences, Livingstone, Zambia

[2]Department of Medicine, Vanderbilt University Medical Center, Nashville, Tennessee, USA.

Emails: Emmanuel L. Luwaya luwayaemmanuel@gmail.com -Lackson Mwape luckyprincemwape5@gmail.com -Kaole Bwalya bwalyakaole60@gmail.com -Chileleko Siakabanze chileleko.siakabanze@gmail.com -Benson M. Hamooya benmalambo@gmail.com -Sepiso K. Masenga sepisomasenga@gmail.com

Address for Correspondence:

Mr. Emmanuel L. Luwaya

Arthur Davison Children's Hospital

P.O Box 240227

Ndola, Zambia

Tel: +260963326481/ +260979886640

Email: luwayaemmanuel@gmail.com

Abstract

Background: An increase in the prevalence of HIV drug resistance (HIVDR) has been reported in recent years, especially in persons on non-nucleoside reverse transcriptase inhibitors (NNRTIs) due to their low genetic barrier to mutations. However, there is a paucity of epidemiological data quantifying HIVDR in the era of new drugs like dolutegravir (DTG) in sub-Saharan Africa. We, therefore, sought to determine the prevalence and correlates of viral load (VL) suppression in adult people with HIV (PWH) on a fixed-dose combination of tenofovir disoproxil fumarate/lamivudine/dolutegravir (TLD) or tenofovir alafenamide/emtricitabine/dolutegravir (TAFED) and describe patterns of mutations in individuals failing treatment.

Methods: We conducted a cross-sectional study among 384 adults living with HIV aged ⩾15 years between May 2023 and August 2023. Demographic, laboratory and clinical data were collected from electronic health records using a data collection form. Viral load suppression was defined as plasma HIV-1 RNA VL of <1000 copies/ml after being on ART for ≥ 6 months. SPSS version 22 to analyze the data. Descriptive statistics and logistic regression were the statistical methods used.

Results: The median (interquartile range (IQR)) age was 22 (IQR 18, 38) years, and 66.1% (n=254) were females. VL suppression was 90.9% (n=349); (95% confidence interval (CI) 87.6%-93.6%) after switching to TLD/TAFED. Among the virally suppressed, the majority (67%, n=234) were female. Those who missed ⩾2 doses in the last 30 days prior to the most recent review were less likely to attain viral suppression compared to those who did not miss any dose (adjusted odds ratio (AOR) 0.075; 95% CI 0.028-0.199; p<0.001), whereas those who were on a protease inhibitor (PI)-based regimen for at least 6 months before switching to TLD/TAFED were also less likely to attain viral suppression compared to those who were on NNRTI-based regimen for at least 6 months before switching; AOR 0.193 (95% CI 0.057-0.652; p=0.008. Four participants had resistance mutations to lamivudine and tenofovir. The most common NRTI mutations were M184MV and K65R while K101E was the most common NNRTI mutation.

Conclusion: Our findings show that viral suppression was high after switching to TLD/TAFED; but lower than the last 95 target of UNAIDS. Adherence to, and type of antiretroviral therapy used before switching to TLD/TAFED were significant correlates of VL suppression. We, therefore, recommend prompt switching of PWH to TLD/TAFED regimen and close monitoring to enhance adherence to therapy.

Commented [MN2]: Double check the ststement

Commented [MN3]: These two parts of the statement are not comparable. The first part of the statement is based on time while the second part of it is based on change in regimen. Therefore, the statement is quite confusing.

Introduction

The United Nations Program on HIV/AIDS (UNAIDS) has set a fast-track goal of 95-95-95 to end the AIDS pandemic by 2030 [1,2]. The goal is for 95% of people with HIV to be aware of their status, 95% of those who are aware should receive treatment, and 95% of those receiving treatment should be HIV virally suppressed [3]. In 2021, 75% of people with HIV knew their status, 85% were receiving treatment, and 68% had viral suppression [4]. While efforts are being made to achieve the target, HIV Drug Resistance (HIVDR) poses a significant threat in the fight against HIV/AIDS [5-8]. There has been an increase in the prevalence of HIVDR in people living with HIV (PLWH), which has been stated as "the emerging threat of HIV drug resistance in Africa" by the World Health Organization (WHO) and UNAIDS [3,9]. Before transition to TLD/TAFED, Zambia reported an estimated 4.3% of individuals on ART for 12-24 months to have some drug resistance [10]. In addition to this, 46.9% of individuals on first-line ART with unsuppressed viral load were reported to have resistance to both Non-Nucleoside Reverse Transcriptase Inhibitors (NNRTIs) and Nucleoside Reverse Transcriptase Inhibitors (NRTIs) [10].

With the increase of HIVDR especially in NNRTIs due to their low genetic barrier to mutations [11-16], WHO recommends the switch to dolutegravir-based first-line regimen in countries where pre-treatment drug resistance to NNRTI reaches the 10% threshold [17]. Dolutegravir (DTG) belongs to the integrase strand transfer inhibitor (INSTI) class of antiretroviral drugs [18]. While drugs in this class are generally highly efficacious, elvitegravir (EVG) and raltegravir (RAL) are susceptible to resistance mutations [1,19-21], leading to virologic failure [22]. DTG however, has shown

superiority with regard to genetic barrier to resistance, even more compared to the NNRTI [23]. This has led to recommendation of the use of tenofovir disoproxil fumarate, lamivudine and dolutegravir (TLD) in a single pill, as first- line treatment of HIV regardless of the patient's viral load [23].

Viral load has been used for a long time now as a predictor of response to treatment [24,25] [24]. Individuals with high viral loads ≥1000 copies/microliters are known to be unsuppressed and are recommended for enhanced adherence counselling (EAC) after which another viral load test is done within three months after the last VL test [3]. If still unsuppressed, an HIV drug resistance test is recommended to determine if there are drug resistance mutations [9]. Although DTG is known to have a high gene barrier to resistance, a recent report from Malawi has shown some resistance after the transition to TLD [26]. In this study, we sought to determine the prevalence and correlates of VL suppression and HIVDR mutations in adult PLWH on a fixed-dose combination of TLD or (TAFED) and describe patterns of mutations in individuals failing treatment.

Methods

Study design and setting

This study was a retrospective cross-sectional study that was conducted at Arthur Davison Children's Hospital (ADCH) in Ndola district, Zambia, among adults aged ⩾15 years, between 5th, June 2023 and 10th, August 2023. ADCH antiretroviral clinic is a family center that offers services to people living with HIV regardless of their age in addition to HIV screening and testing. ADCH is also a referral hospital to the central and northern regions of Zambia. At the time of the study, viral load monitoring was routine as guided by the national guidelines.

Eligibility criteriab

The study included adults on TLD/TAFED aged ≥15 years living with HIV. For HIVDR mutation analysis, participants were excluded if they had failed genotyping test despite having a VL >1000 copies/ml, if they had missing data or had been on treatment for less than six months before transition to TLD/TAFED. In addition, HIV RNA sequences were excluded if the treatment regimen at the time of genotyping was unknown. For viral suppression, participants who were on treatment for less than six months before transition to TLD/TAFED were excluded.

Sample size

Open-source epidemiologic statistics for public health (OpenEpi) online software was used to compute a total sample size of 384 using the maximum estimated frequency of viral load suppression of 50% at a 95% significance level and 80% power in a population of 1000000.

Data collection and study procedures

Socio-demographic, laboratory and clinical data were collected from medical records using a data collection form. The information was abstracted from electronic-based (SmartCare and sequence files) and paper-based (registers and patient files) health information records. Two sets of viral load data were collected; the first set of viral load data was at least 6 months before participants were switched to TLD/TAFED, while the second set of viral load data was collected after at least 3 months of switching to TLD/TAFED. Data collected in this study was entered into the research electronic data capture (REDCap) before analysis.

We employed a systematic random sampling method to abstract patient files. We determined a sampling interval X by dividing the total number of patient files at a facility by the sample size 384 and taking the whole number only by dropping the decimals. The first file for review was selected (N), by randomly selecting a number between 1 and X (sampling interval). Data abstractors selected the Nth file and then proceeded with selecting file N+X as the first consecutive record for review and continued with adding X until the sample size was been reached. If any patient file from the selected list was excluded from the study, the next eligible record fulfilling the inclusion criteria was selected to replace the excluded file.

Research variables and definitions

The primary outcome variable of this study was viral load suppression and the secondary outcome variable was drug resistance mutations, while the independent variables were, current ART regimen, duration on ART, level of education, marital status, age, sex, body mass index (BMI), duration on ART, ART regimen, viral load,

number of doses missed in the last 30 days, history of Tuberculosis (TB), history of opportunistic infection, hypertension, diabetic status, full blood count, CD4 count, creatinine and number of sexual partners as indicated in patient file.

Viral load suppression was defined as a VL <1000 copies/microliter of plasma [26], while HIVDR was defined as the presence of at least one drug resistance-associated mutation (DRM) according to the World Health Organization 2009 Surveillance Drug Resistance Mutation list, using the Stanford Calibrated Population Resistance analysis tool (version 4.1 beta, available at http://hivdb.stanford.edu/).

Viral load was broken down into four categories for analysis; >1000 copies/ml for those who were virally unsuppressed while those who were virally suppressed were categorized as 20 -1000 copies/ml, less 20 copies/ml and Target Not Detected (TND). ART regimen before transition to TLD/TAFED were in categories; NNRTI + NRTI, INSTI-based and PI-based regimens. Meanwhile, level of education was defined in four categories; No education, primary, secondary and tertiary education. Variables with binary outcomes such as diabetes, history of tuberculosis, opportunistic infection and viral suppression were defined by yes for the presence or history of the condition or no for the absence of the condition. Hypertension was also defined as such; yes for hypertensive participants and no for non-hypertensive participants.

Data analysis

Data were imported into SPSS v.22 for analysis. Continuous variables such as age, BMI, hemoglobin and duration on ART were described using medians and interquartile range (IQR). Categorical variables, such as ART regimen, history of TB, viral load

suppression, number of people with drug resistance and sex were described using frequency and percentage distributions. To determine the relationship between two categorical variables such viral suppression and ART regimen, a chi-square analysis was performed. Multivariable logistic regression model was used to determine the contribution of each variable toward the outcome and to control for confounding. The multivariate logistic regression included all independent variables with a p-value of < 0.05 at bivalent analysis and those with biological significance. All associations with a P value <0.05 were considered statistically significant. We used strengthening the reporting of observational studies in epidemiology (STROBE) checklist for all sections (supporting document 1).

Results

Characteristic of the study population

The study included a total of 384 participants, with females being the majority, 66.1% (n=254) and the median age of all participants was 22 years (Interquartile range (IQR), 18, 39). The majority of participants were single (62.8%, n=241), unemployed (87.2%, n=337), attained secondary education (70.1%, n=269). After switching/initiation on TLD/TAFED, 90.9% participants were virally suppressed (349) compared to 69.8% (268) before switching. Table1.

Table 1: Study Socio-demographic and Clinical characteristics		
**Variables**	**Frequency/median**	**Percentage/IQR**
**Age, *years***	22	(18,38)
**Sex**		
Male	130	33.9
Female	254	66.1
**Marital status**		
Married	111	28.9
Single	241	62.8
Separated/divorced	17	4.4
Widowed	15	3.9
**Employment status**		
Formal employment	47	12.2
Unemployed	337	87.2
**Education**		
No education	10	2.6
Primary	53	13.8
Secondary	269	70.1
Tertiary	51	13.3
**BMI, kg/m2**	21	(19,24)
**Hypertension**		
Yes	20	5.2
No	363	94.5
**Diabetes**		
Yes	1	0.3
No	382	99.5
**History of TB**		
Yes	24	6.3
No	358	93.2
**Opportunistic Infection**		
Yes	99	25.8
No	284	74
**Viral Load Suppression**		
Unsuppressed	35	9.1
Suppressed	349	90.9
**ART Regimen Before**		
NNRTI + NRTI	309	80.5
INSTI-Based	23	6.0
PI-Base	52	13.5
**VL Before Switch/Initiation**		
>1000 copies/ml	116	30.2
20-1000 copies/ml	152	39.6
Less than 20 copies/ml	27	7.0
Target Not Detected	88	22.9
**VL After Switch to TLD/TAFED**		
>1000 copies/ml	35	9.1
20-1000 copies/ml	105	27.4
Less than 20 copies/ml	50	13.0
Target Not Detected	194	50.5
**Doses missed in past 30 days**		
**0**	321	83.6
**1**	31	8.1
**≥2**	28	7.3
**Duration on ART (months)**	127	(95,168)
**Hemoglobin**	12.4	(11.075,13.3)

Abbreviations: OPI, opportunistic infection, PI, protease inhibitor, INSTI, integrase strand transfer inhibitor, NNRTI, non-nucleoside reverse transcriptase inhibitor, NRTI, nucleoside reverse transcriptase

Comparison of socio-demographic and clinical factors between unsuppressed and suppressed participants

A higher proportion of individuals attained viral suppression (90.9%, n=349). Factors significantly associated with viral suppression included number of doses missed ($p<0.001$), ART regimen before switching to TLD/TAFED and viral suppression ($p<0.001$), history of OPI ($p<0.001$) and Body Mass Index ($p=0.034$). Table 2.

Table 2. **Socio-demographic and clinical factors sorted according to suppressed status**

Variables	Viral Load Suppression, n (%)		P value
	**Yes, 349 (90.9)**	**No, 35 (9.1)**	
**Age, (*years*)**	22 (18,39)	20 (17,28)	0.145
**Sex**			
Male	115 (33.0)	15 (42.9)	0.238
Female	234 (67.0)	20 (57.1)	
**Marital status**			
Married	105 (30.1)	6 (17.1)	0.162
Not Married	244 (69.9)	29 (82.9)	
**Employment status**			
Formal employment	45 (12.9)	2 (5.7)	0.286
Unemployed	304 (87.1)	33 (94.3)	
**Education level**			
No education	8 (2.6)	2 (5.9)	0.471
Primary	47 (13.5)	6 (17.6)	
Secondary	246 (70.5)	23 (67.7)	
Tertiary	48 (13.4)	3 (8.8)	
**BMI, kg/m2**	21 (18,23)	20 (19,24)	**0.034**
**Hypertension**			
Yes	20 (5.7)	0 (0)	0.238
No	328 (94.3)	35 (100)	
**Diabetes**			
Yes	1 (0.3)	0 (0)	1.00
No	347 (99.7)	35 (100)	
**History of TB**			
Yes	21 (6.1)	3 (8.6)	0.473
No	326 (93.9)	32 (91.4)	
**Opportunistic Infection**			
Yes	81 (23.3)	18 (51.4)	**0.001**
No	267 (76.7)	17 (48.6)	
**ART Regimen**			
NNRTI	290 (83.1)	19 (54.3)	**<0.001**
INSTI-Based	16 (4.6)	7 (20.0)	
PI-Based	43 (12.3)	9 (25.7)	
**Doses missed in past 30 days**			
0	304 (88.1)	17 (48.6)	**<0.001**
1	28 (8.1)	3 (8.6)	
≥2	13 (3.8)	15 (42.9)	
**Duration on ART (months)**	125.5 (95,167.3)	138 (72,192.8)	0.384
**HB**	12.4 (11.3,13.3)	11.4 (10.7,13.2)	0.188

Abbreviations: BMI, body mass index, OPI, opportunistic infection, PI, protease inhibitor, INSTI, integrase strand transfer inhibitor, NNRTI, non-nucleoside reverse transcriptase inhibitor, NRTI, nucleoside reverse transcriptase

Factors associated with viral suppression in logistic regression

In the univariate analysis, participants with a history of opportunistic infections were 71.3% less likely to attain viral suppression compared to those with a history of no opportunistic infection, odds ratio (OR) 0.287; 95% confidence interval (CI) 0.141, 0.581; p=0.001. Meanwhile, participants who had missed ≥2 doses in the last 30 days

were significantly 95.2% less likely to attain viral suppression compared to those who had not missed any dose in the last 30 days, OR 0.048; 95% CI 0.020, 0.581; p <0.001. Participants who were on a PI-based regimen before switching to TLD/TAFED were significantly 85% less likely to attain viral suppression compared to those who were on NNRTI, OR 0.150; 95%CI 0.055, 0.408; p<0.001. Equally, those who were on INSTI-based regimen (AZT/3TC/DTG) before switching to TLD/TAFED, were 68.7% less likely to attain viral suppression compared to those on NNRTI based regimen, OR 0.313; 95%CI 0.133,0.736); p=0.008, Table 3.

In the multivariate analysis, factors that were significantly associated with viral load suppression included ART regimen before switch/initiation and doses missed in the last 30 days. Compared to those who did not miss a dose in the last 30 days, those who missed ≥2 doses in the last 30 days were 93.6% less likely to attain viral suppression and this was statistically significant (p value <0.001 at 95% CI 0.023,0.178). Meanwhile, those who were on PI-based regimen before switching to or initiation on TLD/TAFED were 79.3% less likely to attain Viral suppression compared to those on NNRTI+NTRI based regimen and the results were statistically significant (p value = 0.010, at 95% CI 0.063, 0.687); Table 3.

Table 3. Factors associated with viral suppression in logistic regression

Variables	Univariate OR (95%CI)	P value	AOR (95%CI)	P value
**Age**	1.021 (0.991,1.052)	0.166	1.029 (0.982,1.079)	0.235
**Sex (Male as ref.)**				
**Female**	1.526 (0.754,3.091)	0.240	1.537 (0.612,3.862)	0.361
**History of OPI (No as ref.)**				
**Yes**	0.287 (0.141,0.581)	**0.001**	0.478 (0.185,1.234)	0.127
**Doses missed in the last 30 days (0 as ref.)**				
**> or = 2**	0.048 (0.020,0.118)	**<0.001**	0.064 (0.023,0.178)	**<0.001**
**Regimen before switch/initiation (ref: NNRTI+NRTI)**				
**PI-based (TDF/3TC/LPV-r)**	0.150 (0.055,0.408)	**<0.001**	0.207 (0.063,0.687)	**0.010**
**INSTI-based (AZT/3TC/DTG)**	0.313 (0.133,0.736)	**<0.008**	0.553 (0.183,1.670)	0.294
**Education level (Ref: No Educ.)**				
**Primary**	1.958 (0.335,11.465)	0.456	2.572 (0.306,21.598)	0.384
**Secondary**	2.674 (0.536,13.342)	0.230	5.74 (0.736,44.766)	0.095
**Tertiary**	4.00 (0.575,27.819)	0.161	3.080 (0.233,40.703)	0.975
**Marital Status (Ref: Married)**				
**Unmarried**	0.481 (0.194,1.192)	0.114	0.599 (0.150,2.396)	0.469
**Employment Status (Ref: Employed**				
**Unemployed**	0.409 (0.095,1.765)	0.231	0.965 (0.100,9.306)	0.975
**Duration on ART**	0.997 (0.990,1.004)	0.376	1.000 (0.992,1.008)	0.986

Abbreviations: AZT, Zidovudine, 3TC, lamivudine, LPV-r, liponavir, TDF, tenofovir OPI, opportunistic infection, PI, protease inhibitor, INSTI, integrase strand transfer inhibitor, NNRTI, non-nucleoside reverse transcriptase inhibitor, NRTI, nucleoside reverse transcriptase

Distribution of HIV drug (HIVDR) resistance mutations in samples with any HIVDR.

Overall, the 3 most common mutations were M184MV (50%), K65R (50%), and K101E (50%). There was only one Major PI mutation M46L (25%) while there were two PI-Accessory mutations V32VALP (25%) and L33LF (25%). The most prevalent mutations were NNRTI mutations, accounting for 9 different types of mutations, while there were 5 different types of NRTI mutations (table 4).

**Table 4. Distribution of HIV drug resistance mutations in samples with any HIVDR (n=4)**		
**Drug group**	Mutation	Frequency of mutation, n (%)
**NRTI** (Abacavir, Lamivudine,	M184MV	2 (50)
Emtricitabine)	K65R	2 (50)
	A62AV	1 (25)
	S68N	1 (25)
	T69del	1 (25)
**NNRTI** (Nevirapine, Efavirenz)	K101E	2 (50)
	V179F	1 (25)
	G190S	1 (25)
	A98G	1 (25)
	Y181C	1 (25)
	G190A	1 (25)
	V179VAIT	1 (25)
	K103N	1 (25)
	V106M	1 (25)
**PI Major** (Indinavir)	M46L	1 (25)
**PI Accessory**	V32VALP	1 (25)
	L33LF	1 (25)
NNRTI, Non-nucleoside reverse transcriptase inhibitor, NRTI, nucleoside reverse transcriptase inhibitor, PI, protease inhibitor.		

Discussion

The study provides critical data on viral load suppression and drug resistance mutations in adults taking TLD/TAFED at ADCH, Ndola Zambia. In this study we observed an increase in viral load suppression of participants after switching to or initiation on TLD/TAFED regimen. The second observation was that the number of doses missed by participants was a significant factor in viral suppression. Participants who had missed ≥2 doses in the last 30 days were less likely to attain viral suppression compared to those who did not miss a dose in the last 30 days. Finally, we also observed a low prevalence of HIVDR mutations, 0.103% (n=4), with the pattern of mutations showing NRTI and NNRTI being the most prevalent, 50% each while mutations to PIs and INSTIs were less prevalent and no mutation to dolutegravir were observed.

Our study shows that dolutegravir-based regimens are more efficacious and increase level of viral load suppression as it has been reported in other studies [26]. We observed that viral suppression was maintained in most participants, especially those who were previously on NNRTI+NRTI combination such as Tenofovir, Lamivudine, Efavirenz (TLE) and Atripla (Tenofovir, Emtricitabine, Efavirenz), and even increased after switching to TLD/TAFED. However, TLD/TAFED have shown superiority in increasing viral suppression and will be key factor in attaining the UNAID target of 95%-95%-95%. This justifies the decision to transition PLWH to TLD/TAFED regardless of their VL. There may be concerns however, that individuals who have high viraemia before switch/initiation to TLD/TAFED are likely to experience viral failure. We observed that of the participants with viral failure after switching to TLD/TAFED, some had a VL >1000 copies/ml before switching and these participants were later reported to have

HIV drug resistance mutations after switching to TLD/TAFED. This observation is consistent with other studies where a high viral load before transition is described as a risk for viral failure [24]. Additionally, the high prevalence of HIVDR mutations to NNRTI and NRTI as proven even in other studies, like our study, supports the Zambia consolidated HIV guidelines on the use of dolutegravir which has a high genetic barrier to resistance. This is of more significant because NRTI mutations such as K65R, confer high resistances to tenofovir and lamivudine which are used as backbone for TLD [27]. These mutations were prevalent among individuals failing treatment in our study population. A recent study in Malawi reported treatment-emergent resistance to dolutegravir in two individuals who among few others had viral load ≥50 copies/m and had NRTI resistance at the time of transition to TLD of which both of them had resistance to both lamivudine and tenofovir. Although our study did not include genetic resistance testing before switching to TLD/TAFED, it is worth noting that all the four individuals that had HIVDR mutations had a VL ≥1000 copies/ml before switching to TLD/TAFED regimen.

Commented [MN4]: Check grammar……significance

The TLD was recommended by WHO as preferred first-line ART regimen for adolescents and adults due to high genetic barrier to resistance that dolutegravir confers [23] and our findings support the existing evidence of viral suppression attained on TLD with the use of lamivudine and tenofovir disoproxil fumarate as backbone, as compared to NNRTI-based regimen. TAFED is another preferred combination that is commonly used in adults. While drug potency is vital, poor adherence is a fundamental factor to treatment failure in most cases. Our findings show correlation between the number of doses missed and viral suppression, which is in agreement with the

ADVANCE and NAMSAL studies in which it was determined that adherence contributes to viral suppression [28,29]. With adherence being a significant factor in viral suppression, there is need to re-evaluate the adherence monitoring methods. The current method applied in our set up at ADCH is based on client disclosure (self-reporting) during the review, which is less objective compared to measuring drug concentration in plasma or urine that can provide more accurate adherence information. With self-reporting, healthcare personnel have to rely on the information given by the client to determine whether or not they have been adherent which makes self-reporting an unreliable method of monitoring adherence. To help manage clients with regard to adherence, review of all those that report to not have missed any dose follow the regular pharmacy schedule of 3 months while those that report to have missed one dose are scheduled for review monthly. Individuals that reported to have missed two or more doses are scheduled for 4 weeks of weekly appointment visits to ensure that they are adhering to treatment. While this may be helpful in some instances still falls short of proper monitoring of adherence. As emphasized in other studies, we reemphasize the need of coming up with proper ways of monitoring treatment adherence of PWLH as this has proven to be an important factor in viral suppression and HIV drug resistance mutations [30].

Study limitations and strengths

Our study did not include genetic resistance testing before switching to TLD/TAFED. Because of that, we could not establish whether the mutations in the participants were present before switch or developed after switching to the TLD/TAFED regimen. Our

study was also limited to one study center, and therefore might not give an accurate picture of the prevalence of viral load suppression and especially HIV-1 drug resistance mutations in Zambia.

Our findings support government's effort to transition adult PLWH to TLD/TAFED in order to attain viral suppression and also gives an understanding of the factors associated with viral suppression. With our large sample size, the findings are a good representation of the study population.

Conclusion

The study showed an increase in the prevalence of HIV viral suppression after switch from other regimens to dolutegravir based regimen, TLD/TAFED. The number of doses missed by a patient and the regimen before switching correlated with viral suppression. Furthermore, we observed a low prevalence of HIV drug resistance in participants after 6 months of switching to TLD/TAFED. While there were no drug resistance mutations to dolutegravir observed, NNRTI and NRTI mutations which confer resistance to tenofovir and lamivudine were observed among the few individuals that had HIVDR mutations. Based on our findings, we recommend the re-evaluation of adherence monitoring methods in order to improve patient management and enhance viral suppression.

Abbreviations

ADR, Acquired HIV drug resistance; ART, Antiretroviral therapy; ARV, Antiretroviral (drugs); ATV/r, Atazanavir/ritonavir; CI, Confidence interval; DBS, Dried blood spot;

DRM Drug resistance mutation; DRV/r, Darunavir/ritonavir; DTG, Dolutegravir; GAP, Global Action Plan on HIV drug resistance; HIVDR, HIV drug resistance; LMIC, Low- and middle-income countries; NNRTI, Non-nucleoside reverse-transcriptase inhibitor; NRTI, Nucleoside reverse-transcriptase inhibitor; PDR, Pre-treatment HIV drug resistance; PEPFAR, United States President's Emergency Plan for AIDS Relief; PI, Protease inhibitor; PLWHIV, People living with HIV; TAFED, Tenofovir Alafenamide, Emtricitabine and Dolutegravir; TLD, Tenofovir disproxil fumarate, Lamivudine and Dolutegravir; UNAIDS, Joint United Nations Program on HIV/AIDS; VL, Viral load; WHO, World Health Organization

Declarations

Ethics approval and consent to participate

Ethical approval was obtained from Mulungushi University Health Sciences Research Ethics Committee (MUHSREC) (Assurance No. FWA0002888 IRB00012281 of IORG0010344 on the 5th of April, 2023 and the National Health Research Authority (NHRA) on the 11th May, 2023. Permission to conduct research was given by Arthur Davison children's hospital administration. Written consent was waived by the ethics committee due to the retrospective nature of the study where secondary data was used. No personal identifying data were obtained such that it was not possible to identify participants.

Consent for publication

Not applicable

Availability of data and materials

All data generated or analyzed during this study are included in this published article. For other data, these may be requested through the corresponding author.

Competing interests

The authors declare that they have no competing interests

Funding

No funding was received for this research project.

Author's contributions

SKM and ELL conceived the study. SKM, BMH, LM and ELL contributed to the writing of the manuscript. ELL conducted data collection. SKM, BMH, KB, CS, LM conducted data validation, visualization and analysis. ELL is the principal investigator. SKM is the senior author and guarantor. All authors read, provided feedback, and approved the final manuscript.

Acknowledgments

We are very grateful to all Laboratory personnel and the Senior Medical Superintendent's office at Arthur Davison Children's Hospital (ADCH) for their support. We would also like to thank the ADCH ART clinic staff for their continued support and assistance during the data collection process.

5.0 References

1. Stover J, Bollinger L, Izazola JA, Loures L, DeLay P, Ghys PD. What Is Required to End the AIDS Epidemic as a Public Health Threat by 2030? The Cost and Impact of the Fast-Track Approach. PLoS ONE. 2016;11: e0154893. doi:10.1371/journal.pone.0154893

2. HIV_DrugRes_FINAL_01_04_19_online.pdf. Available: https://www.afro.who.int/sites/default/files/2019-04/HIV_DrugRes_FINAL_01_04_19_online.pdf

3. Heath K, Levi J, Hill A. The Joint United Nations Programme on HIV/AIDS 95–95–95 targets: worldwide clinical and cost benefits of generic manufacture. AIDS. 2021;35: S197. doi:10.1097/QAD.0000000000002983

4. Frescura L, Godfrey-Faussett P, Feizzadeh A. A, El-Sadr W, Syarif O, Ghys PD. Achieving the 95 95 95 targets for all: A pathway to ending AIDS. PLoS ONE. 2022;17: e0272405. doi:10.1371/journal.pone.0272405

5. Hudson FP, Mulenga L, Westfall AO, Warrier R, Mweemba A, Saag MS, et al. Evolution of HIV-1 drug resistance after virological failure of first-line antiretroviral therapy in Lusaka, Zambia. Antivir Ther. 2019;24: 291–300. doi:10.3851/IMP3299

6. Lepik KJ, Harrigan PR, Yip B, Wang L, Robbins MA, Zhang WW, et al. Emergent drug resistance with integrase strand transfer inhibitor-based regimens. AIDS. 2017;31: 1425. doi:10.1097/QAD.0000000000001494

7. Sörstedt E, Carlander C, Flamholc L, Hejdeman B, Svedhem V, Sönnerborg A, et al. Effect of dolutegravir in combination with Nucleoside Reverse Transcriptase Inhibitors (NRTIs) on people living with HIV who have pre-existing NRTI mutations. Int J Antimicrob Agents. 2018;51: 733–738. doi:10.1016/j.ijantimicag.2018.01.009

8. Gupta RK, Jordan MR, Sultan BJ, Hill A, Davis DH, Gregson J, et al. Global trends in antiretroviral resistance in treatment-naive individuals with HIV after rollout of antiretroviral treatment in resource-limited settings: a global collaborative study and meta-regression analysis. Lancet. 2012;380: 1250–1258. doi:10.1016/S0140-6736(12)61038-1

9. Bertagnolio S, Beanland RL, Jordan MR, Doherty M, Hirnschall G. The World Health Organization’s Response to Emerging Human Immunodeficiency Virus Drug Resistance and a Call for Global Action. J Infect Dis. 2017;216: S801–S804. doi:10.1093/infdis/jix402

10. Zulu PM, Toeque M-G, Hachaambwa L, Chirwa L, Fwoloshi S, Siwingwa M, et al. Retrospective Review of Virologic and Immunologic Response in Treatment-

Experienced Patients on Third-Line HIV Therapy in Lusaka, Zambia. J Int Assoc Provid AIDS Care. 2021;20: 23259582211022463. doi:10.1177/23259582211022463

11. Group TTS. Global epidemiology of drug resistance after failure of WHO recommended first-line regimens for adult HIV-1 infection: a multicentre retrospective cohort study. Lancet Infect Dis. 2016;16: 565. doi:10.1016/S1473-3099(15)00536-8

12. Kiekens A, Dierckx de Casterlé B, Pellizzer G, Mosha IH, Mosha F, Rinke de Wit TF, et al. Exploring the mechanisms behind HIV drug resistance in sub-Saharan Africa: conceptual mapping of a complex adaptive system based on multi-disciplinary expert insights. BMC Public Health. 2022;22: 455. doi:10.1186/s12889-022-12738-4

13. World Health Organization. Global report on early warning indicators of HIV drug resistance: technical report. Geneva: World Health Organization; 2016. Available: https://apps.who.int/iris/handle/10665/246219

14. Arimide DA, Amogne MD, Kebede Y, Balcha TT, Adugna F, Ramos A, et al. High Level of HIV Drug Resistance and Virologic Nonsuppression Among Female Sex Workers in Ethiopia: A Nationwide Cross-Sectional Study. J Acquir Immune Defic Syndr 1999. 2022;89: 566–574. doi:10.1097/QAI.0000000000002908

15. World Health Organization. HIV drug resistance strategy. 2021 update. Geneva: World Health Organization; 2021. Available: https://apps.who.int/iris/handle/10665/343175

16. World Health Organization. HIV drug resistance strategy. 2021 update. Geneva: World Health Organization; 2021. Available: https://apps.who.int/iris/handle/10665/343175

17. World Health Organization. Consolidated guidelines on person-centred HIV strategic information: strengthening routine data for impact. Geneva: World Health Organization; 2022. Available: https://apps.who.int/iris/handle/10665/360948

18. Dorward J, Lessells R, Drain PK, Naidoo K, de Oliveira T, Pillay Y, et al. Dolutegravir for first-line antiretroviral therapy in low-income and middle-income countries: uncertainties and opportunities for implementation and research. Lancet HIV. 2018;5: e400–e404. doi:10.1016/S2352-3018(18)30093-6

19. Underwood M, Horton J, Nangle K, Hopking J, Smith K, Aboud M, et al. Integrase Inhibitor Resistance Mechanisms and Structural Characteristics in Antiretroviral Therapy-Experienced, Integrase Inhibitor-Naive Adults with HIV-1 Infection Treated with Dolutegravir plus Two Nucleoside Reverse Transcriptase Inhibitors in the DAWNING Study. Antimicrob Agents Chemother. 66: e01643-21. doi:10.1128/AAC.01643-21

20. World Health Organization, UNAIDS. Integration of mental health and HIV interventions: key considerations. Geneva: World Health Organization; 2022. Available: https://apps.who.int/iris/handle/10665/353571

21. Keene CM, Griesel R, Zhao Y, Gcwabe Z, Sayed K, Hill A, et al. Virologic efficacy of tenofovir, lamivudine and dolutegravir as second-line antiretroviral therapy in adults failing a tenofovir-based first-line regimen. AIDS Lond Engl. 2021;35: 1423–1432. doi:10.1097/QAD.0000000000002936

22. Ndashimye E, Avino M, Olabode AS, Poon AFY, Gibson RM, Li Y, et al. Accumulation of integrase strand transfer inhibitor resistance mutations confers high-level resistance to dolutegravir in non-B subtype HIV-1 strains from patients failing raltegravir in Uganda. J Antimicrob Chemother. 2020;75: 3525–3533. doi:10.1093/jac/dkaa355

23. Rolle C-P, Berhe M, Singh T, Ortiz R, Wurapa A, Ramgopal M, et al. Dolutegravir/lamivudine as a first-line regimen in a test-and-treat setting for newly diagnosed people living with HIV. AIDS. 2021;35: 1957. doi:10.1097/QAD.0000000000002979

24. Bernal E, Gómez JM, Jarrín I, Cano A, Muñoz A, Alcaraz A, et al. Low-Level Viremia Is Associated With Clinical Progression in HIV-Infected Patients Receiving Antiretroviral Treatment. JAIDS J Acquir Immune Defic Syndr. 2018;78: 329–337. doi:10.1097/QAI.0000000000001678

25. Ehrenkranz PD, Baptiste SL, Bygrave H, Ellman T, Doi N, Grimsrud A, et al. The missed potential of CD4 and viral load testing to improve clinical outcomes for people living with HIV in lower-resource settings. PLoS Med. 2019;16: e1002820. doi:10.1371/journal.pmed.1002820

26. Schramm B, Temfack E, Descamps D, Nicholas S, Peytavin G, Bitilinyu-Bangoh JE, et al. Viral suppression and HIV-1 drug resistance 1 year after pragmatic transitioning to dolutegravir first-line therapy in Malawi: a prospective cohort study. Lancet HIV. 2022;9: e544–e553. doi:10.1016/S2352-3018(22)00136-9

27. Carnimeo V, Pulido Tarquino IA, Fuentes S, Vaz D, Molfino L, Tamayo Antabak N, et al. High level of HIV drug resistance informs dolutegravir roll-out and optimized NRTI backbone strategy in Mozambique. JAC-Antimicrob Resist. 2021;3: dlab050. doi:10.1093/jacamr/dlab050

28. Castillo-Mancilla JR, Haberer JE. Adherence Measurements in HIV: New Advancements in Pharmacologic Methods and Real-Time Monitoring. Curr HIV/AIDS Rep. 2018;15: 49–59. doi:10.1007/s11904-018-0377-0

29. Mudhune V, Gvetadze R, Girde S, Ndivo R, Angira F, Zeh C, et al. Correlation of Adherence by Pill Count, Self-report, MEMS and Plasma Drug Levels to Treatment Response Among Women Receiving ARV Therapy for PMTCT in Kenya. AIDS Behav. 2018;22: 918–928. doi:10.1007/s10461-017-1724-7

30. McCluskey SM, Pepperrell T, Hill A, Venter WDF, Gupta RK, Siedner MJ. Adherence, resistance, and viral suppression on dolutegravir in sub-Saharan Africa: implications for the TLD era. AIDS Lond Engl. 2021;35: S127-S135. doi:10.1097/QAD.0000000000003082

Supporting Information

Click here to access/download
Supporting Information
STROBE_checklist_v4_cross-sectional.pdf

Other

Click here to access/download
Other
SMHS-MU1-2023-38 Ethical Clearance.pdf

Other

Click here to access/download
Other
NHRA Response.pdf

Other

Click here to access/download
Other
ViralLoadSuppression_DATA_LABELS_2023-07-02_1240.xlsx
