# Supplementary material for: Viral load suppression and HIV-1 drug resistance mutations in persons with HIV on TLD/TAFED in Zambia
Source: PLoS One. 2024 Sep 6;19(9):e0308869. doi: 10.1371/journal.pone.0308869 (PMC11379217; doi:10.1371/journal.pone.0308869)
Supplement: Attachment — Submitted filename: Response to Reviewers.docx [file pone.0308869.s007.docx]

Mulungushi University,

P.O Box 60009,

Livingstone.

22^nd^ July 2024.

The Reviewers,

PLOS ONE Journal.

Dear PLOS ONE reviewers,

**RE: RESPONSE TO REVIEWER COMMENTS**

Thank you so much for reviewing our manuscript and providing invaluable reviews. We appreciate your review comments which we believe have improved our manuscript's readability and credibility.

We have taken time to correct our manuscript and provided a manuscript copy with track changes to reflect our work on the manuscript.

Below are our responses to the review comments.

When submitting your revision, we need you to address these additional requirements.

1. Please ensure that your manuscript meets PLOS ONE's style requirements, including those for file naming. The PLOS ONE style templates can be found at 
<https://journals.plos.org/plosone/s/file?id=wjVg/PLOSOne_formatting_sample_main_body.pdf> and 
<https://journals.plos.org/plosone/s/file?id=ba62/PLOSOne_formatting_sample_title_authors_affiliations.pdf>

**Response from authors:** Thank you. We have referred to the comments and have since adjusted the naming of the manuscript and supporting documents to PLOS ONE’s style requirements.

2. We note that your Data Availability Statement is currently as follows: All relevant data are within the manuscript and its supporting information files.

Please confirm at this time whether or not your submission contains all raw data required to replicate the results of your study. Authors must share the “minimal data set” for their submission. PLOS defines the minimal data set to consist of the data required to replicate all study findings reported in the article, as well as related metadata and methods (<https://journals.plos.org/plosone/s/data-availability#loc-minimal-data-set-definition>).

**Response from authors:** Thank you, we have since attached the raw data to this submission.

3. When completing the data availability statement of the submission form, you indicated that you will make your data available on acceptance. We strongly recommend all authors decide on a data sharing plan before acceptance, as the process can be lengthy and hold up publication timelines. Please note that, though access restrictions are acceptable now, your entire data will need to be made freely accessible if your manuscript is accepted for publication. This policy applies to all data except where public deposition would breach compliance with the protocol approved by your research ethics board. If you are unable to adhere to our open data policy, please kindly revise your statement to explain your reasoning and we will seek the editor's input on an exemption. Please be assured that, once you have provided your new statement, the assessment of your exemption will not hold up the peer review process.

**Response from authors:** Thank you, we have since attached the raw data to this submission.

4. Please amend either the title on the online submission form (via Edit Submission) or the title in the manuscript so that they are identical.

**Response from authors:** Thank you very much. The title on the online submission has been edited to make identical to the title in the manuscript.

5. Your ethics statement should only appear in the Methods section of your manuscript. If your ethics statement is written in any section besides the Methods, please move it to the Methods section and delete it from any other section. Please ensure that your ethics statement is included in your manuscript, as the ethics statement entered into the online submission form will not be published alongside your manuscript.

**Response from authors:** Thank you, we have since deleted the ethics statement in other parts of the manuscript and only retained it in the methods section.

6. Please include captions for your Supporting Information files at the end of your manuscript, and update any in-text citations to match accordingly. Please see our Supporting Information guidelines for more information: <http://journals.plos.org/plosone/s/supporting-information>.

**Response from authors:** Thank you, we have since made the captions as per recommendation.

7. Please review your reference list to ensure that it is complete and correct. If you have cited papers that have been retracted, please include the rationale for doing so in the manuscript text, or remove these references and replace them with relevant current references. Any changes to the reference list should be mentioned in the rebuttal letter that accompanies your revised manuscript. If you need to cite a retracted article, indicate the article’s retracted status in the References list and also include a citation and full reference for the retraction notice.

**Response from authors:** Thank you, we have reviewed our reference list as recommended. We have not cited any retracted article.

**Review Comments to the Author**

Reviewer 1
1. It’s mentioned that “In 2021, 75% of people with HIV knew their status, 85% were receiving treatment, and 68% had viral suppression according to a review by Frescura” The percentages seem low, is this the current situation in Zambia as of 2023 when the study was carried out?

**Response from authors:** Thank you very much. We have made the revisions and included the correct percentages from the Zambia Population-based HIV and AIDS Impact Assessment (ZAMPHIA) survey conducted between May-December 2021. This is the latest country-wide data showing that Zambia has improved. Among adults (15 years and older) in Zambia living with HIV, 88.7 percent were aware of their status, 98.0 percent of those who knew their status were on ART, and 96.3 percent of those on ART achieved viral load suppression..

2. Provide more literature on levels of HIVDR in the DTG era apart from Malawi…what is the situation in other countries especially Zambia? Are there no other studies that have reported resistance to DTG in Zambia? When was DTG adopted in Zambia?

**Response from authors:** Thank you so much. We have revised and included the period of adoption of DTG. We’ve also included literature from other countries on DTG. However, according to our knowledge, there are no studies that have reported DTG resistance in Zambia.

Reviewer 2
1. Correct the dates of carrying out the study is it between 5th, June 2023 and 10th, August 2023 or between May 2023 and August 2023 “

**Response from author:** Thank you for the comment. We have corrected the dates of carrying out of the study, which is between 5^th^ June, 2023 and 10^th^ August, 2023

2. For sample size calculation, what guided a viral load suppression of 50%? Is the national rate?

**Response from authors:** Thank you so much for the comment. The 50% was used to help obtain the maximal sample size from our study population since national rate was not well documented

3. What guided the virally suppressed categories of 20 -1000 copies/ml, less 20 copies/ml and Target Not Detected (TND).

**Response from authors:** Thank you very much. The categories were guided by the result output of the viral load instrument that was used. The machine gives result such Target not detected (TND) and less than 20 copies/ml. So the categorization was only for descriptive purposes that provides more data around those that were virally suppressed. However, since the outcome variable “viral load suppression” was categorized as a binary variable, where viral suppression categories included “virally suppressed <1000 copies/ml” and “unsuppressed viral load >1000 copies/ml”, we have now used these categories in the manuscript for consistency.

Under results
1. What drugs made up the NNRTI +NRTI regimen?

**Response from author:** Thank you so much. The NNRTI + NRTI combination was made up of Tenofovir + Efavirenz + Lamivudine/Emtricitabine. We have since revised and included this description in our results.

2. Were all the 384 participants non-suppressed?

**Response from authors:** Thank you so much. Not all participants were non-suppressed. Only 116 participants were non-suppressed before switching to DTG and only 35 were non-suppressed after switching to DTG

3. How were the OR for viral suppression after switching to DTG calculated?- it appears that these were calculated as if all participants were non-suppressed before switching, therefore giving the impression that PI-based regimen were 85% less likely to suppress after switching, something which is not consistent with the body of literature in this area. See the numbers of the different regimens in table one and two.

**Response from authors:**

The OR for the Regimen before switching to DTG-based regimen was calculated for all participants regardless of viral suppression status before switching to DTG. This is because some participants who were virally suppressed before switching became unsuppressed after at last 6 months on DTG suggesting an interplay of other factors contributing to viral suppression. Therefore, to account for changes occurring even in those who were suppressed during the period on DTG, we included them in the analysis.

We have recalculated the OR only for those who were unsuppressed (as shown below), and the multivariable results are not very different in terms of statistical significance. However, neither of the specific regimen were associated with viral suppression in the multivariable model either because the sample size was not highly powered for subgroup analysis or because of confounding factors that have been included in the multivariable regression model. PI based regimen were not significantly associated with viral suppression even on univariable analysis with this change.

| **Variables** | **Univariate OR (95%CI)** | **P value** | **AOR (95%CI)** | **P value** |
| --- | --- | --- | --- | --- |
| Regimen before switch/initiation (ref: NNRTI+NRTI) |  |  |  |  |
| PI-based  (TDF/3TC/LPV-r) | 0.405 (0.130,1.262) | 0.119 | 0.777 (0.193, 3.135) | 0.723 |
| INSTI-based  (AZT/3TC/DTG) | 0.135 (0.029,0.627) | **0.011** | 0.350 (0.050, 2.476) | 0.293 |
| Note: other variables in the model are not shown here | | | | |

4. Table is redundant since number of those with HIVDR mutation is just 4 and its distribution is described in the section before the table

**Response from authors:** Thank you very much for the review. We have revised and removed the table.

We want to thank the reviewers again for taking time to review and make suggestions that has improved our manuscript. We now hope it is acceptable for publication.

Yours,

Emmanuel Luwaya

On behalf of the authors
